# Synthesis of Multiple Emission Carbon Dots from Dihydroxybenzoic Acid via Decarboxylation Process

**DOI:** 10.3390/nano13142062

**Published:** 2023-07-13

**Authors:** Pengfei Li, Jijian Xu, Ziye Shen, Wenning Liu, Li An, Dan Qu, Xiayan Wang, Zaicheng Sun

**Affiliations:** Center of Excellence for Environmental Safety and Biological Effects, Beijing Key Laboratory for Green Catalysis and Separation, Department of Chemistry, Beijing University of Technology, 100 Pingleyuan, Beijing 100124, China; lipf05034@emails.bjut.edu.cn (P.L.); xujijian2023@163.com (J.X.); shenziye20210531@163.com (Z.S.); lwnbjut@163.com (W.L.); 08131@bjut.edu.cn (L.A.); danqu@bjut.edu.cn (D.Q.); xiayanwang@bjut.edu.cn (X.W.)

**Keywords:** carbon dots, multicolor, dihydroxybenzoic acid, photocatalysis

## Abstract

Carbon dots (CDs), as a new zero-dimensional carbon-based nanomaterial with desirable optical properties, exhibit great potential for many application fields. However, the preparation technique of multiple emission CDs with high yield is difficult and complex. Therefore, exploring the large-scale and straightforward synthesis of multicolor CDs from a simple carbon source is necessary. In this work, the solvent-free method prepares a series of multicolor emission CDs from dihydroxybenzoic acid (DHBA). The maximum emission wavelengths are 408, 445, 553, 580, and 610 nm, respectively, covering the visible light region. The 2,4- and 2,6-CDs possess the longer emission wavelength caused by the 2,4-, and 2,6-DHBA easily undergo decarboxylation to form the larger sp^2^ domain graphitized structure. These CDs incorporated with g-C_3_N_4_ can significantly improve the photocatalytic water-splitting hydrogen production rate by extending the visible light absorption and enhancing the charge separation efficiency. The long-wavelength emission CDs can further enhance photocatalytic activity primarily by improving visible light absorption efficiency.

## 1. Introduction

Carbon dots (CDs) as an emerging nanomaterial attracted great interest due to their high stability, easy modification, good biocompatibility, excellent optical properties, distinctive electronic features, etc. [1,2,3,4,5] Among them, the tunable and excellent optical properties play a significant part in many advanced application fields, such as monochromatic light-emitting diodes (LEDs), fluorescence imaging, fluorescence probe, information encryption, etc. [6,7,8,9]. Sun’s group prepared red, green, and blue emission CDs through thermal pyrolysis of citric acid and urea. Additionally, these CDs were fabricated as multiple-color and white LEDs, which serve as a low-cost alternative phosphor of LEDs [10]. Qu et al. synthesized high photoluminescence quantum yield (PL QY) and near-infrared (NIR) emission CDs for NIR fluorescence bioimaging in vivo. These CDs with distinguished photostability, biocompatibility, and high contrast show great potential for ideal NIR probes [11]. Lin et al. reported long-lived room temperature phosphorescence (RTP) CDs could be used for time-resolved bioimaging, information encryption, and storage [12]. Therefore, plenty of researchers are devoted to the luminescence regulation of CDs and the synthesis of multicolor CDs.

Generally, the synthetic strategies of CDs can be divided into top-down and bottom-up ways. The structure of CDs prepared through the top-down method is simple and unregulated, leading the optical properties of CDs to be regulated hard. The bottom-up approach is used chiefly to prepare multiple emission CDs [13,14,15]. Jiang et al. first reported the preparation of multicolor CDs (red, green, and blue) with o-phenylenediamine (oPD), m-phenylenediamine, and p-phenylenediamine as precursors via the facile solvothermal method [16]. Since then, more and more researchers have successfully regulated the CDs’ emissions through hydrothermal and solvothermal processes. However, the yield of CDs is usually poor and insufficient to meet the application requirement entirely. In addition, the solvent molecule may participate in the reaction in the forming process of CDs, which contributes to the forming process of CDs becoming more complex and harder to analyze. The solvent-free synthetic method not only can increase the CDs’ yield but also avoid solvent molecules’ interference, which has been proposed as an ideal strategy for preparing CDs. For instance, Li et al. synthesized red emission CDs with high yield from oPD and catechol (CAT) through the solvent-free method, successfully revealing the PL mechanism and forming process [17]. The aluminum chloride hexahydrates (AlCl_3_·6H_2_O) as the catalyst can not only disperse the precursors but also promote dehydration and carbonization.

CDs attracted great interest in the photocatalytic field due to their high stability, easy modification, tunable spectral absorption, distinctive electronic features, and other excellent properties. It can be incorporated with C_3_N_4_ to form CDs@C_3_N_4_ composite photocatalysts and provide a variety of functions in the C_3_N_4_ photocatalytic process, such as photosensitizers, spectral converters, electron reservoirs, etc. Sun and coworkers prepared a new metal-free g-C_3_N_4_@CDs composite. CDs act as electron mediators, promoting the photogenerated electron by the rapid transfer of g-C_3_N_4_ to the CDs with 96% electron transfer efficiency, significantly facilitating the generation of H_2_ under visible light irradiation [3]. Tang’s group constructed a g-C_3_N_4_@CDs heterostructure through a simple one-step hydrothermal method. The photoresponse range of this metal-free photocatalyst is expanded from the UV to the NIR region to generate H_2_ from water splitting [18]. Lu et al. explored the contributions of different kinds of nitrogen chemical states by doping CDs to promote g-C_3_N_4_ photocatalytic hydrogen production. Their finding suggested that pyrrole-N-doping CDs promote the highest hydrogen production rate of g-C_3_N_4_ by promoting photocarrier separation [19]. However, CDs process excellent and adjustable optical properties, corresponding to different photoelectric properties. That may have a different influence on the photocatalytic performance of g-C_3_N_4_.

Herein, we use the isomer of dihydroxybenzoic acid (DHBA; 3,4-DHBA, 2,5-DHBA, 3,5-DHBA, 2,6-DHBA, 2,4-DHBA) as the precursor synthesized multicolor fluorescent CDs via the solvent-free method with high yield. The 2,4-DHBA and 2,6-DHBA are more likely to undergo dihydroxylation to form a large graphitization structure and particle size, leading to a red-shift of emission and absorption. The graphitization structure and particle size of 3,4-CDs, 2,5-CDs, 3,5-CDs, 2,6-CDs, and 2,4-CDs increase gradually, and the maximum emission wavelength reaches 385, 445, 553, 580, and 610 nm, respectively. Then, these CDs are mixed with urea and sinter to form a series of g-C_3_N_4_@CDs composites via a pyrolysis method. These CDs, as photosensitizers, can broaden the absorption region and promote the CN photocatalytic activity. The g-C_3_N_4_ modified by 3,4-CDs, 2,5- CDs, 3,5-CDs, 2,6-CDs, and 2,4-CDs exhibit a gradually rising hydrogen production rate. The 2,4-CDs can maximally improve the absorption of the composite photocatalyst in the visible region and promote the separation of the photocarrier on the surface. Its H_2_ production rate is 3.60 times higher than that of pure CN.

## 2. Materials and Methods

### 2.1. Reagents and Materials

All the chemicals were used as received without any further treatment. The 2,4-dihydroxybenzoic acid (2,4-DHBA, >99%), urea (>99%), and ethanol (>99%) were purchased from Aladdin. The 2,5-DHBA (>99%), 2,6-DHBA (>99%), and 3,5-DHBA (>99%) were purchased from Macklin. The 3,4-DHBA, triethanolamine, and chloroplatinic acid were purchased from Bide Pharmatech (Shanghai, China), Damao (Tianjin, China), and Sigma-Aldrich (Shanghai, China), respectively.

### 2.2. Preparation of the Five CDs and CDs@C_3_N_4_ Composite Photocatalyst

All five CDs were prepared through the solvent-free method. The processes are as follows: 1.0 g (8.20 mmol) of each of the following, 2,4-DHBA, 2,6-DHBA, 3,5-DHBA, 2,5-DHBA, and 3,4-DHBA, were mixed with 0.1 g (0.41 mmol) of AlCl_3_·6H_2_O, respectively, and ground to uniform using an agate mortar. Then, these mixtures were transferred to a 25 mL Teflon-lined autoclave and heated for 12 h at 220 °C. After the autoclave cooled to room temperature naturally, the raw product was dissolved in an ethanol solution and centrifuged at 10,000 rpm/min to remove the over-carbonization production. The solution was dialyzed in the 3500 cut-off dialysis bag against the mixed solution of DI water and ethanol by 1:1 until the fluorescence of the dialyzate disappeared. Finally, the CDs’ powders were obtained via vacuum freeze-drying.

The CDs@C_3_N_4_ composite photocatalyst was synthesized via a simple mixed thermal pyrolysis method. The processes are as follows: 25 mg of CDs were mixed well with 25 g of urea and transferred to an alumina crucible. Subsequently, it was heated in the muffle furnace at 5 °C/min to 550 °C for 3 h, forming a series of gray CDs@C_3_N_4_ powder.

### 2.3. Photocatalytic Hydrogen Evolution

The photocatalytic water-splitting hydrogen evolution was reacted in a closed Pyrex glass system (Beijing Perfectlight Technology Co., Ltd., Labsolar-6A, Beijing, China). Generally, 50 mg of 1.0 wt% Pt-loaded composite photocatalyst was dispersed in a 100 mL 10% triethanolamine (TEOA) aqueous solution. A 300 W Xe lamp (Beijing Perfectlight Technology Co., Ltd., Labsolar-6A, Beijing, China) with the 420 nm cut-off filter was used as the light source and adjusted to 500 mW cm^−1^. The temperature was controlled as constant at 10 °C via a circulation cooling water method. The amount of hydrogen produced was determined by gas chromatography (Shimadzu GC-2014 C).

## 3. Results and Discussion

### 3.1. The Optical Properties of CDs

**Figure 1:**
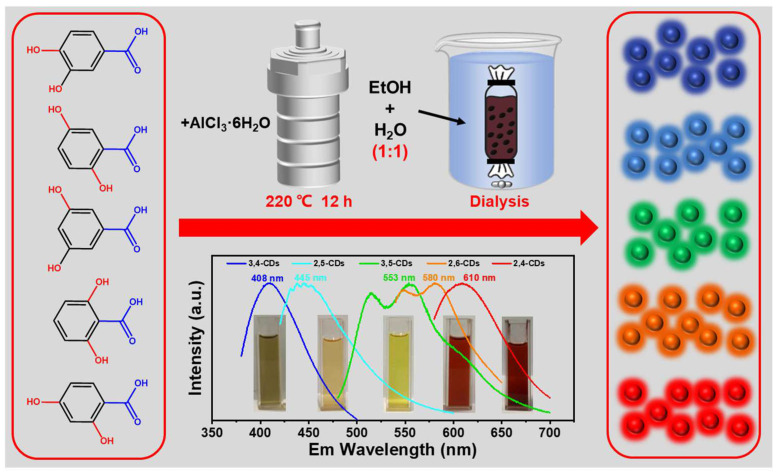
A solvent-free synthesis of the multicolor fluorescent CDs from 2,4-DHBA, 2,6-DHBA, 3,5-DHBA, 2,5-DHBA, 3,4-DHBA with the catalysis of AlCl3·6H2O. Also represented is the maximum emission wavelength spectra of the 2,4-CDs, 2,6-CDs, 3,5-CDs, 2,5-CDs, and 3,4-CDs, respectively.

The multicolor emission CDs are synthesized through the solvent-free method at 220 °C, and with AlCl_3_·6H_2_O as a catalyst. After dialysis in the mixture of ethanol and water (1:1), dark blue-, light blue-, green-, orange-, and red-color emission CDs (2,4-CDs, 2,6-CDs, 3,5-CDs, 2,5-CDs, 3,4-CDs) are obtained, which correspond to 2,4-DHBA, 2,6-DHBA, 3,5-DHBA, 2,5-DHBA, 3,4-DHBA as precursors, respectively (Figure 1). These as-obtained CDs and ethanol solutions exhibit different clear color appearances under daylight. These CDs’ maxima photoluminescence (PL) emission wavelengths are measured as 408, 445, 553, 580, and 610 nm, respectively, covering the whole visible light region.

**Figure 2:**
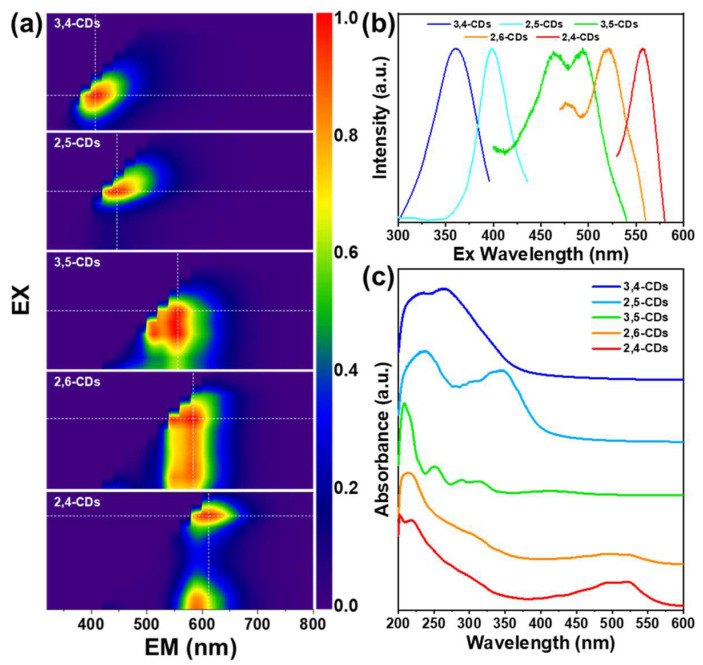
(**a**) Excitation–Emission Matrix (EEM) spectra of 2,4-CDs, 2,6-CDs, 3,5-CDs, 2,5-CDs, 3,4-CDs dissolved in ethanol. (**b**) The corresponding PL excitation spectra of the five types of CDs dissolved in ethanol. (**c**) The corresponding UV-vis spectra of the five types of CDs dissolved in ethanol.

In the excitation–emission matrix (EEM) spectra of these CDs in ethanol (Figure 2a), the 3,4-CDs and 2,5-CDs exhibit the simple and sole PL emission center at 407 nm and 446 nm, respectively, which cover the blue light region. The corresponding PL excitation wavelengths are 360 nm and 400 nm (Figure 2b). The 3,5-CDs EEM spectra show the 514 nm and 553 nm double PL emission center from the excitation of 500 nm. The 2,6-CDs also process the dual PL emission center at 550 nm and 580 nm, excited by the excitation range from 400 nm to 580 nm. The 2,4-CDs EEM spectra depict two PL emission centers at 612 nm excited by 560 nm, and 590 nm excited by 400 nm. The UV-vis absorption spectra of the five types of CDs in Figure 2c show strong absorption at 200–250 nm but different absorption at longer-wavelength regions. The absorption band at 263 nm of 3,4-CDs and 236 nm and 348 nm bands of 2,5 CDs assign to the π-π* transitions of the C=C and C=O bands, which generate the dark blue and light blue light FL emission [20]. The 3,5-CDs appear as distinct absorption bands at 375–450 nm, indicating different kinds of surface state transitions [10,21,22]. The 2,6-CDs and 2,4-CDs exhibit similar absorption bands at 450–550 nm, which leads to longer wavelength FL emission.

### 3.2. The Structure Properties of CDs

**Figure 3:**
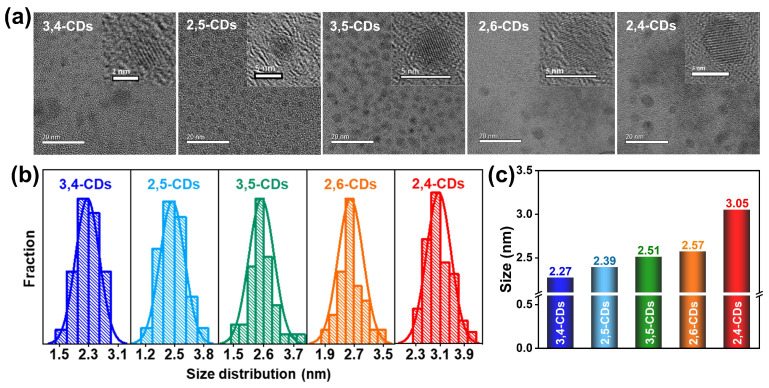
(**a**) TEM and HRTEM images (inset) of 3,4-CDs, 2,5-CDs, 3,5-CDs, 2,6-CDs, and 2,4-CDs. (**b**) Particle size distribution diagrams of these CDs. (**c**) The histogram of the average particle size of these five CDs.

Transmission electron microscopy (TEM) images of these CDs are presented in Figure 3a, showing these CDs are uniform-dispersed and homogeneous nanodots. The high-resolution (HR) TEM provided in the insets illustrates all these CDs exhibit good crystalline carbon structure. Lattice fringe distance is observed at 0.21 nm, attributed to the (100) in-plane graphene lattice. The particle sizes of CDs all follow the normal distribution and increase gradually with the emission wavelength red-shift (Figure 3b). The average diameters of 3,4-CDs, 2,5-CDs, 3,5-CDs, 2,6-CDs, and 2,4-CDs are 2.27, 2.39, 2.51, 2.57, and 3.05 nm, respectively (Figure 3c). This result suggests the multiple optical properties of these CDs may be caused by the different graphitization degrees and assorted sizes.

**Figure 4:**
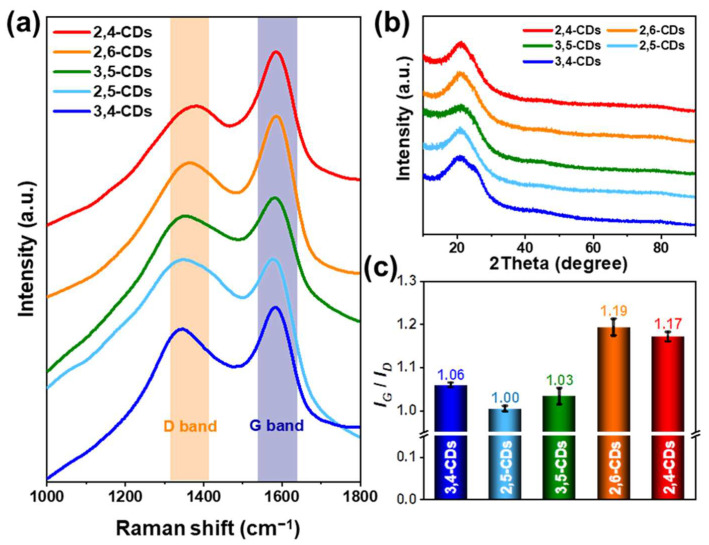
(**a**) Raman spectra of 2,4-CDs, 2,6-CDs, 3,5-CDs, 2,5-CDs, 3,4-CDs. (**b**) X-ray diffraction (XRD) pattern of these five CDs. (**c**) The ratio diagrams of ID/IG of these five CDs.

The laser confocal Raman microspectroscopy (Figure 4a) and the X-ray diffraction (XRD) pattern (Figure 4b) are carried out to appraise the graphene structure of these five CDs. The Raman spectra of these five CDs reveal similar peak shapes at 1360 cm^−1^ (D band) and 1580 cm^−1^ (G band), but the 2,4-CDs and 2,6-CDs exhibit higher intensity at the G band. Different forms of graphene can be identified by the D and G bands. The D band characterizes the amorphous or defective carbon structure, and the G band reflects the in-plane vibration of the sp^2^ graphitic carbon [23]. The intensity ratios of *I_G_*/*I_D_* are 1.06, 1.00, 1.03, 1.19, and 1.17 for 3,4-, 2,5-, 3,5-, 2,6-, and 2,4-CDs, respectively (Figure 4c). That indicates the graphitization degree of 2,6- and 2,4-CDs is much higher than the other three CDs. The XRD pattern (Figure 4b) depicts a similar peak at 21°, corresponding to the (002) graphitic structure. However, the peak indicates d spacing is 0.42 nm and larger than the regular d spacing of 0.35 nm of the graphitic (002) crystal plane, which is caused by the functional groups in the layer surface or edge [24].

**Figure 5:**
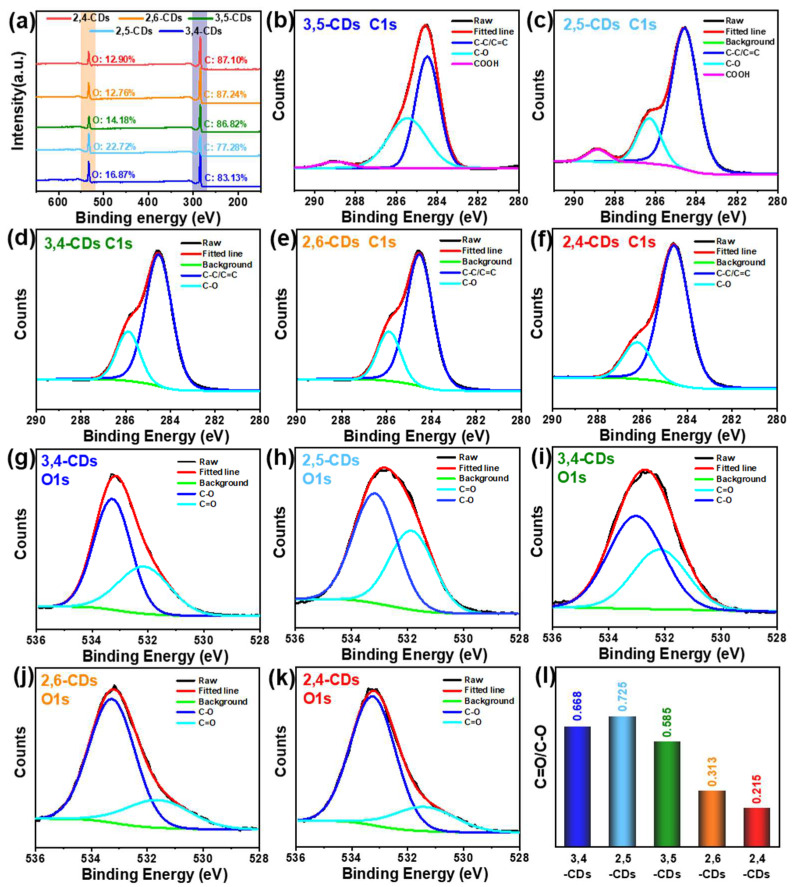
(**a**) Full-scale XPS survey spectrum of the five CDs. (**b**–**g**) The high-resolution C1s XPS spectrum of 3,5-, 2,5-, 3,4-, 2,6-, and 2,4-CDs, respectively. (**h**–**l**) The high-resolution O1s XPS spectrum of 3,5-, 2,5-, 3,4-, 2,6-, and 2,4-CDs, respectively. (**m**) The distribution of the C=O/C-C content ratios of the five CDs.

X-ray photoelectron spectroscopy (XPS) is used to further analyze the compositions of the five CDs, which contribute to clarifying the different forming processes. Full-scan XPS spectra (Figure 5a) of the five CDs show two characteristic peaks at 285 and 513 eV, corresponding to C1s and O1s, respectively, and the oxygen content of 2,4- and 2,6-CDs (12.90%, 12.76%) much lower than that of 3,5-, 2,5- and 3,4-CDs (14.18%, 22,72%, 16.87%). In the C1s high-resolution XPS spectra of 3,5- and 2,5-CDs (Figure 5b,c), the spectra can be deconvoluted into three binding energy peaks: C=C/C-C (284.6 eV), C-O (286.0 eV) and -COOH (288.8 eV). However, the 3,4-, 2,6- and 2,4-CDs C1s spectra can only be deconvoluted into the C=C/C-C and C-O binding energy peaks (Figure 5d–f) [25]. Additionally, the proportion of the C-O peaks area gradually decreases with the CDs’ emission wavelength red-shift. That may be the critical factor causing the different optical properties of the five CDs. In the O1s spectra (Figure 5g–k), these CDs exhibit two binding energy peaks at C-O (284.6 eV) and C=O (286.0 eV), but the C-O content of 2,6- and 2,4-CDs is significantly lower than the other three CDs [26]. As shown in Figure 5l, the C=O/C-C content ratios of 3,4-, 2,5-, 3,5-, 2,6-, and 2,4-CDs are 0.668, 0.725, 0.585, 0.313, and 0.215, respectively. That is consistent with the C1s spectra, demonstrating the longer-wavelength emission CDs contain less carboxyl.

### 3.3. The Fluorescence Mechanism and Forming Process of CDs

**Scheme 1:**
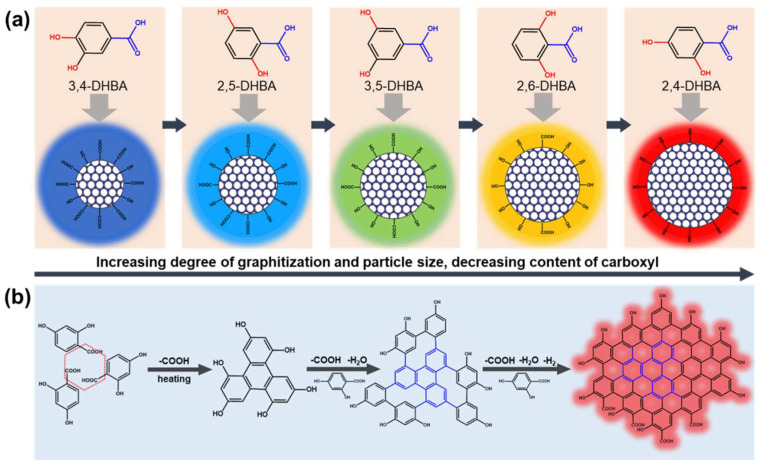
A possible (**a**) PL mechanism for CDs with tunable PL emission and (**b**) formation mechanism of 2,4-CDs.

In the well-embraced theory of CDs’ PL mechanism, the optical properties depend on particle size and effective conjugation length (degree of graphitization) [27]. The π orbital increases and overlaps with the effective conjugation length enlarging, gradually, and leading to the orbital energy level difference between the highest occupied molecular orbital (HOMO) and lowest unoccupied molecular orbital (LUMO) of the CDs’ reduction and the emission wavelength red-shift. These conform to the experiment results. Therefore, the PL mechanism of these CDs should belong to the carbon core state. The particle size and degree graphitization of 3,4-, 2,5-, 3,5-, 2,6-, and 2,4-CDs increase in turn, gradually contributing to the maximum emission wavelength red-shift (Scheme 1a).

In addition, there has been a trend in 3,4-, 2,5-, 3,5-, 2,6-, and 2,4-CDs toward the lower carboxyl content, which provides a basis and idea for speculating the forming process of these kinds of CDs. Previous research about the benzene ring electronic structure of phenolic compounds has shown that the decarboxylated reactivity of DHBA is mainly dependent on different net charges (q_1_–q_7_) between the benzene ring carbon atom (C_1_) attached to a carboxyl group and the carboxyl carbon atom (C_7_) [28]. The calculation results show that the ortho- and para-hydroxyl groups can reduce the decarboxylation temperature of hydroxybenzoic acid. The meso-hydroxyl group may produce a higher thermal decarboxylation temperature [29]. Based on these results and previous reports, a possible model of the formation process and mechanism is proposed as an example of 2,4-DHBA, shown in Scheme 1b. At the initial reaction stage, the adjacent 2,4-DHBA molecules start decarboxylating and begin the coupling reaction at high temperature and high pressure. This could be considered a building block due to their smallest sp^2^ domain [7]. With the increasing reaction temperature and time, these intermediate building blocks continued to react with the 2,4-DHBA via decarboxylation coupling and dehydration reaction. Subsequently, the intermediate cross-linking polymer carbonizes in situ with deamination and dehydrogenation reactions, generating the large conjugated sp^2^ cluster with -OH and -COOH [30]. Decarboxylation is essential to produce the larger effective conjugation length CDs. That is why 2,4-DHBA and 2,6-DHBA can generate long-wavelength emission CDs. However, the cross-linking process is relatively challenging, the reason for that being the steric hindrance of 2,6-DHBA, leading to the emission wavelength of 2,6-CDs being slightly more blue-shifted than 2,4-CDs.

### 3.4. The Facilitated Photocatalysis of g-C_3_N_4_ by CDs

**Figure 6:**
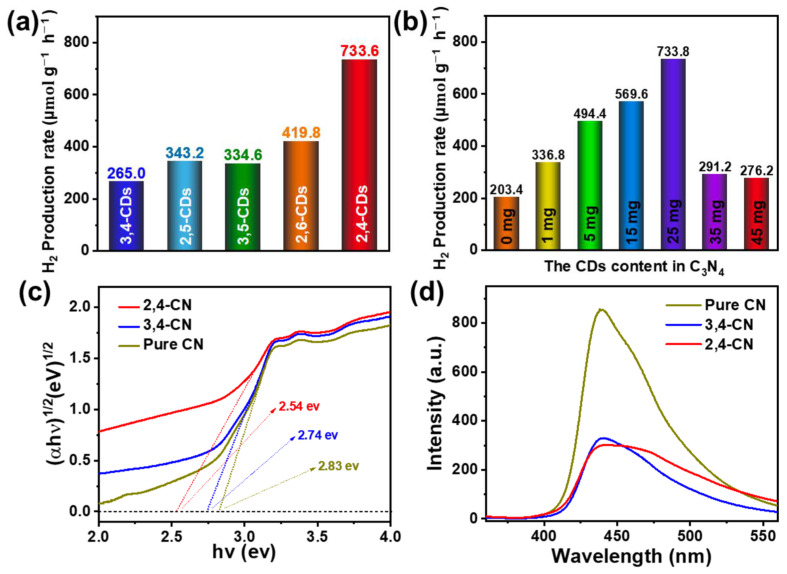
H_2_ production rate of (**a**) different CDs@C_3_N_4_ composite photocatalysts and (**b**) the different 2,4-CDs content in the composite photocatalyst. (**c**) The Kubelka–Munk function of pure CN, 2,4- and 3,4-CN. (**d**) PL spectra of pure CN, 2,4- and 3,4-CN under 340 nm excitation.

Subsequently, the five CDs are mixed with urea and undergo thermal pyrolysis to obtain five kinds of the CDs@C_3_N_4_ (3,4-CN, 2,5-CN, 3,5-CN, 2,6-CN, and 2,4-CN) composite photocatalyst. TEM microscopy of the typical 2,4-CN is shown in Figure S1. The composite exhibits a flaky and ultrathin structure similar to pure CN. The HRTEM offers about 5 nm CDs with graphite structure attachments on the CN flake. The XRD pattern (Figure S2) exhibits similar peaks at 12.9° and 27.8°, which assign to the (210) orthorhombic plane and interlayer stacking (002) facet of CN, respectively, suggesting the interposition of CDs does not destroy the original structure of pure CN [31]. The XPS spectra (Figure S3) show that the composite material has higher oxygen content than pure C_3_N_4_. In the high-resolution O1s XPS spectrum, the relative amount of C=O of composite material is significantly higher than the pure C_3_N_4_, indicating the CDs may be connected with C_3_N_4_ via the C=O bonds. Figure 6a presents the optimized photocatalytic H_2_ production activity of this series of composite photocatalysts. Compared with the H_2_ production rate of 203.4 μmol g^−1^ h^−1^ for pure CN, the photocatalytic activity of 3,4-CN, 2,5-CN, and 3,5-CN increased to some extent by about 1.5 times. Additionally, the H_2_ production rates of 2,6-CN and 2,4-CN significantly enhance to 419.8 and 733.6 μmol g^−1^ h^−1^, respectively, which are 2.06 and 3.60 times higher than pure CN. The photocatalysis hydrogen production efficiency of a similar composition is listed in Table S1. Compared to other materials, the 2,4-CDs@C_3_N_4_ exhibit relatively high H_2_ yield, and the CDs show a significant promoting effect. Subsequently, the CDs’ compound quantity in C_3_N_4_ has been optimized, as shown in Figure 6b and Figure S4a. The photocatalytic H_2_ production activity positively correlates with the CDs’ dose effect in the 0–25 mg range. As soon as the content of CDs exceeds 25 mg, the H_2_ production rate drops markedly to 291.2 μmol g^−1^ h^−1^, which may be occasioned by the extra CDs destroying the ordered structure of C_3_N_4_, affecting the photogenerated charge transfer efficiency. The photocatalytic activity of the 2,4-CN composite photocatalyst does not exhibit a distinct decline in the four continuous 5 h of the H_2_ production reaction cycle (Figure S4b), implying the composite photocatalyst exhibits excellent photochemical stability.

To illuminate the effect of CDs in the photocatalytic H_2_ production process, the UV-vis DRS (Figure S5) and PL spectra (Figure 6d) of pure CN, 2,4-, and 3,4-CN were carried out to analyze the light absorption capacity and charge separation efficiency. As shown in Figure S5, the absorption of pure CN is concentrated in the region before 450 nm, the 2,4-, and 3,4-CN display a tail absorption, and the absorption of 2,4-CN after 420 nm is a more obvious enhancement. Figure 6c presents the corresponding Kubelka–Munk function. The optical band gaps are 2.83, 2.74, and 2.54 eV for pure CN, 3,4-CN, and 2,4-CN, respectively. The band gap of 2,4-CN is significantly more reduced than pure CN and pure CN. In the PL spectra (Figure 6d), the fluorescence intensity of 2,4- and 3,4-CN are reduced considerably more than pure CN, but no intensity difference between the 2,4- and 3,4-CN are observed. That means the charge separation efficiencies of 2,4- and 3,4-CN are almost equal but significantly higher than pure CN. The possible promoting mechanism of different optical properties of CDs from the same system is presented based on the above reasons. The CDs can extend the visible light absorption and enhance the charge separation efficiency. Furthermore, the long-wavelength emission CDs are the most effective because they can maximally promote the photocatalyst’s absorption capacity. The proposed mechanism of the photocatalyst is shown in Figure S6. Under visible light excitation, CDs as photosensitizers are excited to generate photogenerated electrons, which transfer to g-C_3_N_4_ to reduce the hydrogen ions in water to produce H_2_.

## 4. Conclusions

In summary, a series of multicolor CDs are successfully obtained from dihydroxybenzoic acid through a solvent-free method. The maximum fluorescence emission wavelengths of 3,4-, 2,5-, 3,5-, 2,6-, and 2,4-CDs are gradually red-shifted, which can cover the visible spectrum. The particle size and graphitization degree (sp^2^ domain) increase the significant factors in tuning the optical properties of these CDs. During the formation of these CDs, the 2,4- and 2,6-DHBA undergo decarboxylation much easier to form the most stable sp^2^ building block and further extend to the larger sp^2^ graphitized structure. These CDs can promote the photocatalytic activity of g-C_3_N_4_ by expanding the visible light absorption and enhancing the charge separation efficiency. Additionally, the 2,4-CN shows the highest photocatalytic activity due to the long-wavelength emission CDs more effectively extending the visual light absorption quality of g-C_3_N_4_.

## Data Availability

The data presented in this study are available on request from the corresponding author.

## Supplementary Materials

The following supporting information can be downloaded at https://www.mdpi.com/article/10.3390/nano13142062/s1, Figure S1: Representative TEM image of CDs@C_3_N_4_ composite photocatalyst under the different; Figure S2: XRD pattern of pure C_3_N_4_ and CDs@C_3_N_4_ composite material; Figure S3: XPS survey spectrum of the (a) pure C_3_N_4_, and (b) composite material; Figure S4: (a) Photocatalytic H_2_ production of 2,4-CN with different content CDs, loading 1.0 wt% Pt in the 10 vol% triethanolamine (TEOA) under light irradiation (λ > 420 nm). (b) The stability experiment of the best composite material; Figure S5: DRS UV-vis spectra of pure CN, 2,4- and 3,4-CN. Figure S6: The schematic diagram of the composite photocatalytic hydrogen generation mechanism; Table S1: The photocatalysis hydrogen production efficiency comparative table of CDs composition. References [32,33,34,35,36,37,38,39,40,41,42] are cited in the Supplementary Materials.

## References

[1] Zheng M., Ruan S., Liu S., Sun T., Qu D., Zhao H., Xie Z., Gao H., Jing X., Sun Z. (2015). Self-Targeting Fluorescent Carbon Dots for Diagnosis of Brain Cancer Cells. ACS Nano.

[2] Qu D., Zheng M., Du P., Zhou Y., Zhang L., Li D., Tan H., Zhao Z., Xie Z., Sun Z. (2013). Highly Luminescent S, N Co-doped Graphene Quantum Dots with Broad Visible Absorption Bands for Visible Light Photocatalysts. Nanoscale.

[3] Qu D., Liu J., Miao X., Han M., Zhang H., Cui Z., Sun S., Kang Z., Fan H., Sun Z. (2018). Peering into Water Splitting Mechanism of g-C_3_N_4_-carbon Dots Metal-free Photocatalyst. Appl. Catal. B.

[4] Li D., Jing P., Sun L., An Y., Shan X., Lu X., Zhou D., Han D., Shen D., Zhai Y. (2018). Near-Infrared Excitation/Emission and Multiphoton-Induced Fluorescence of Carbon Dots. Adv. Mater..

[5] Bao X., Yuan Y., Chen J., Zhang B., Li D., Zhou D., Jing P., Xu G., Wang Y., Hola K. (2018). In Vivo Theranostics with Near-infrared-emitting Carbon Dots-highly Efficient Photothermal Therapy Based on Passive Targeting after Intravenous Administration. Light Sci. Appl..

[6] Qu D., Yang D., Sun Y., Wang X., Sun Z. (2019). White Emissive Carbon Dots Actuated by the H-/J-Aggregates and Forster Resonance Energy Transfer. J. Phys. Chem. Lett..

[7] Ding H., Zhou X.-X., Zhang Z.-H., Zhao Y.-P., Wei J.-S., Xiong H.-M. (2021). Large Scale Synthesis of Full-color Emissive Carbon Dots from a Aingle Carbon Source by a Solvent-free Method. Nano Res..

[8] Qu S., Wang X., Lu Q., Liu X., Wang L. (2012). A Biocompatible Fluorescent Ink Based on Water-soluble Luminescent Carbon Nanodots. Angew. Chem. Int. Ed. Engl..

[9] Zheng M., Xie Z., Qu D., Li D., Du P., Jing X., Sun Z. (2013). On-off-on Fluorescent Carbon Dot Nanosensor for Recognition of Chromium(VI) and Ascorbic Acid Based on the Inner Filter Effect. ACS Appl. Mater. Interfaces.

[10] Miao X., Qu D., Yang D., Nie B., Zhao Y., Fan H., Sun Z. (2018). Synthesis of Carbon Dots with Multiple Color Emission by Controlled Graphitization and Surface Functionalization. Adv. Mater..

[11] Liu Y., Lei J.H., Wang G., Zhang Z., Wu J., Zhang B., Zhang H., Liu E., Wang L., Liu T.M. (2022). Toward Strong Near-Infrared Absorption/Emission from Carbon Dots in Aqueous Media through Solvothermal Fusion of Large Conjugated Perylene Derivatives with Post-Surface Engineering. Adv. Sci..

[12] Jiang K., Hu S., Wang Y., Li Z., Lin H. (2020). Photo-Stimulated Polychromatic Room Temperature Phosphorescence of Carbon Dots. Small.

[13] Niu X., Song T., Xiong H. (2021). Large Scale Synthesis of Red Emissive Carbon Dots Powder by Solid State Reaction for Fingerprint Identification. Chin. Chem. Lett..

[14] Jiang K., Wang Y., Gao X., Cai C., Lin H. (2018). Facile, Quick, and Gram-Scale Synthesis of Ultralong-Lifetime Room-Temperature-Phosphorescent Carbon Dots by Microwave Irradiation. Angew. Chem. Int. Ed. Engl..

[15] Zhu Z., Cheng R., Ling L., Li Q., Chen S. (2020). Rapid and Large-Scale Production of Multi-Fluorescence Carbon Dots by a Magnetic Hyperthermia Method. Angew. Chem. Int. Ed. Engl..

[16] Jiang K., Sun S., Zhang L., Lu Y., Wu A., Cai C., Lin H. (2015). Red, Green, and Blue Luminescence by Carbon Dots: Full-Color Emission Tuning and Multicolor Cellular Imaging. Angew. Chem. Int. Ed. Engl..

[17] Li P., Xue S., Sun L., Zong X., An L., Qu D., Wang X., Sun Z. (2022). Formation and Fluorescent Mechanism of Red Emissive Carbon Dots from o-phenylenediamine and Catechol System. Light Sci. Appl..

[18] Xia X., Deng N., Cui G., Xie J., Shi X., Zhao Y., Wang Q., Wang W., Tang B. (2015). NIR Light Induced H_2_ Evolution by a Metal-Free Photocatalyst. Chem. Commun..

[19] Zhang S., Gao M., Zhai Y., Wen J., Yu J., He T., Kang Z., Lu S. (2022). Which Kind of Nitrogen Chemical States Doped Carbon Dots Loaded by g-C_3_N_4_ is the Best for Photocatalytic Hydrogen Production. J. Colloid. Interface. Sci..

[20] Ding H., Yu S.B., Wei J.S., Xiong H.M. (2016). Full-Color Light-Emitting Carbon Dots with a Surface-State-Controlled Luminescence Mechanism. ACS Nano.

[21] Lyu B., Li H.-J., Xue F., Sai L., Gui B., Qian D., Wang X., Yang J. (2020). Facile, Gram-scale and Eco-friendly Synthesis of Multicolor Graphene Quantum Dots by Thermal-driven Advanced Oxidation Process. Chem. Eng. J..

[22] Sun M., Liang C., Tian Z., Ushakova E.V., Li D., Xing G., Qu S., Rogach A.L. (2019). Realization of the Photostable Intrinsic Core Emission from Carbon Dots through Surface Deoxidation by Ultraviolet Irradiation. J. Phys. Chem. Lett..

[23] Verma N.C., Yadav A., Nandi C.K. (2019). Paving the Path to the Future of Carbogenic Nanodots. Nat. Commun..

[24] Yan F., Jiang Y., Sun X., Wei J., Chen L., Zhang Y. (2020). Multicolor Carbon Dots with Concentration-tunable Fluorescence and Solvent-affected Aggregation States for White Light-emitting Diodes. Nano Res..

[25] Li D., Liang C., Ushakova E.V., Sun M., Huang X., Zhang X., Jing P., Yoo S.J., Kim J.G., Liu E. (2019). Thermally Activated Upconversion Near-Infrared Photoluminescence from Carbon Dots Synthesized via Microwave Assisted Exfoliation. Small.

[26] Zhang Q., Wang R., Feng B., Zhong X., Ostrikov K.K. (2021). Photoluminescence Mechanism of Carbon Dots: Triggering High-color-purity Red Fluorescence Emission Through Edge Amino Protonation. Nat. Commun..

[27] Xue S., Li P., Sun L., An L., Qu D., Wang X., Sun Z. (2023). The Formation Process and Mechanism of Carbon Dots Prepared from Aromatic Compounds as Precursors: A Review. Small.

[28] Shangxian Y., Jiangnan G., Xiaoyuan F., Juhao X. (1998). Synthesis and photosensitivity of tung oil-resorcin resin. J. Photopolym. Sci. Technol..

[29] Jiangnan G., Xiao T., Yingquan Z., Shaoren H., Shangxian Y. (1999). Reactivity Determination of Carbon Atoms on Benzene Rings of Phenols. Proc. SPIE.

[30] Jia H., Wang Z., Yuan T., Yuan F., Li X., Li Y., Tan Z., Fan L., Yang S. (2019). Electroluminescent Warm White Light-Emitting Diodes Based on Passivation Enabled Bright Red Bandgap Emission Carbon Quantum Dots. Adv. Sci..

[31] Ma Z., Zong X., Hong Q., Niu L., Yang T., Jiang W., Qu D., An L., Wang X., Kang Z. (2022). Electrostatic potential of the incorporated asymmetry molecules induced high charge separation efficiency of the modified carbon nitride copolymers. Appl. Catal. B.

[32] Sun C., Xu Q., Xie Y., Ling Y., Jiao J., Zhu H., Zhao J., Liu X., Hu B., Zhou D. (2017). High-efficient One-pot Synthesis of Carbon Quantum Dots Decorating Bi_2_MoO_6_ Nanosheets Heterostructure with Enhanced Visible-light Photocatalytic Properties. J. Alloys Compd..

[33] Tian J., Leng Y., Zhao Z., Xia Y., Sang Y., Hao P., Zhan J., Li M., Liu H. (2015). Carbon Quantum dots/hydrogenated TiO_2_ Nanobelt Heterostructures and Their Broad Spectrum Photocatalytic Properties under UV, Visible, and Near-infrared Irradiation. Nano Energy.

[34] Zhang Z., Zheng T., Xu J., Zeng H., Zhang N. (2017). Carbon Quantum Dots/Bi_2_WO_6_ Composites for Efficient Photocatalytic Pollutant Degradation and Hydrogen Evolution. Nano.

[35] Li S., Hu S., Jiang W., Liu Y., Zhou Y., Liu J., Wang Z. (2018). Facile Synthesis of Cerium Oxide Nanoparticles Decorated Flower-like Bismuth Molybdate for Enhanced Photocatalytic Activity toward Organic Pollutant Degradation. J. Colloid. Interf. Sci..

[36] Gogoi D., Koyani R., Golder A.K., Peela N.R. (2020). Enhanced Photocatalytic Hydrogen Evolution using Green Carbon Quantum Dots Modified 1-D CdS Nanowires under Visible Light Irradiation. Sol. Energy.

[37] Liu J., Liu Y., Liu N., Han Y., Zhang X., Huang H., Lifshitz Y., Lee S.-T., Zhong J., Kang Z. (2015). Metal-free Efficient Photocatalyst for Stable Visible Water Splitting via a Two-electron Pathway. Science.

[38] Li L., Zhu X. (2018). Enhanced Photocatalytic Hydrogen Evolution of Carbon Quantum Dot Modified 1D Protonated Nanorods of Graphitic Carbon Nitride. ACS Appl. Nano Mater..

[39] Xie Z., Yu S., Fan X.-B., Wei S., Yu L., Zhong Y., Gao X.-W., Wu F., Zhou Y. (2021). Wavelength-sensitive Photocatalytic H_2_ Evolution from H_2_S Splitting over g-C_3_N_4_ with S,N-codoped Carbon Dots as The Photosensitizer. J. Energy Chem..

[40] Naseri A., Samadi M., Pourjavadi A., Moshfegh A.Z., Ramakrishna S. (2017). Graphitic Carbon Nitride (g-C_3_N_4_)-based Photocatalysts for Solar Hydrogen Generation: Recent Advances and Future Development Directions. J. Mater. Chem. A.

[41] Wang Q., Yang Z. (2016). Industrial Water Pollution, Water Environment Treatment, and Health Risks in China. Environ. Pollut..

[42] Ajmal A., Majeed I., Malik R.N., Idriss H., Nadeem M.A. (2014). Principles and, Mechanisms of Photocatalytic Dye Degradation on TiO_2_ Based Photocatalysts: A Comparative Overview. RSC Adv..

