# Supplementary material for: Synthesis of Multiple Emission Carbon Dots from Dihydroxybenzoic Acid via Decarboxylation Process
Source: Nanomaterials (Basel). 2023 Jul 13;13(14):2062. doi: 10.3390/nano13142062 (PMC10383124; doi:10.3390/nano13142062)
Supplement: Supplementary file 1 [file nanomaterials-13-02062-s001.zip › nanomaterials-2478704-supplementary.pdf]

Supplementary materials

Synthesis of Multiple Emission Carbon Dots from Dihydroxybenzoic Acid via Decarboxylation Process

Pengfei Li, Jijian Xu, Ziye Shen, Wenning Liu, Li An, Dan Qu, Xiayan Wang and Zaicheng Sun*

Center of Excellence for Environmental Safety and Biological Effects, Beijing Key Laboratory for Green Catalysis and Separation, Department of Chemistry, Beijing University of Technology, Beijing 100124, People’s Republic of China.

Email: sunzc@bjut.edu.cn

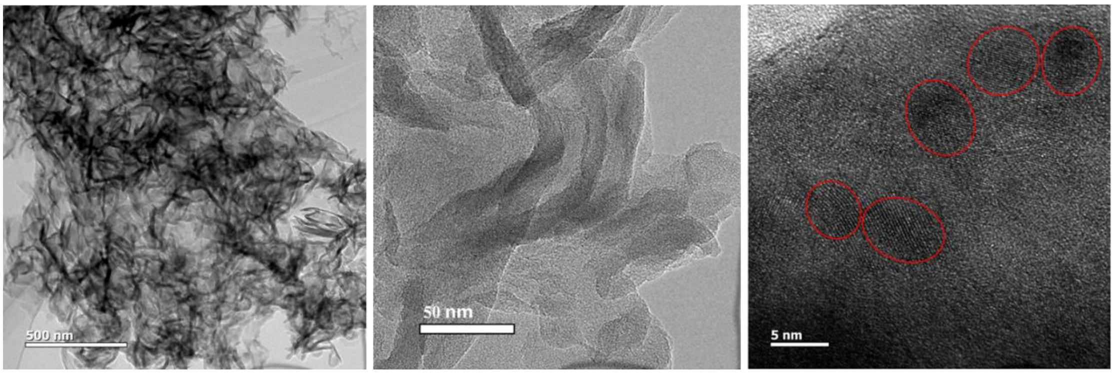

Figure S1. Representative TEM image of CDs@C_3N_4 composite photocatalyst under the different scales.

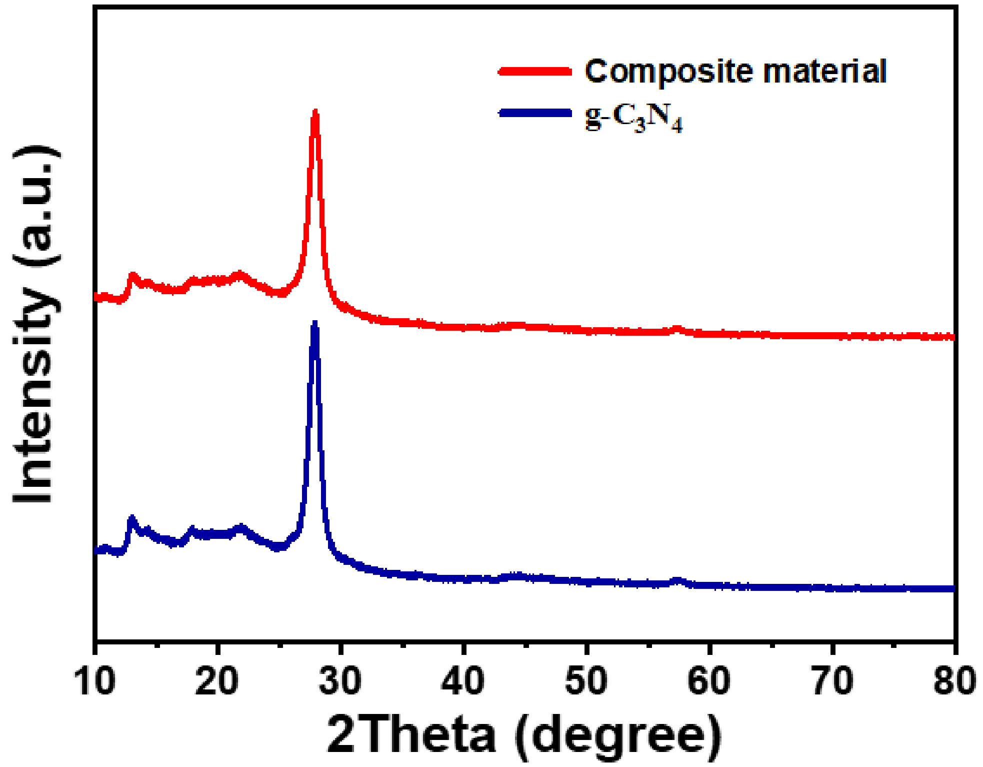

Figure S2. XRD pattern of pure C_3N_4 and CDs@C_3N_4 composite material.

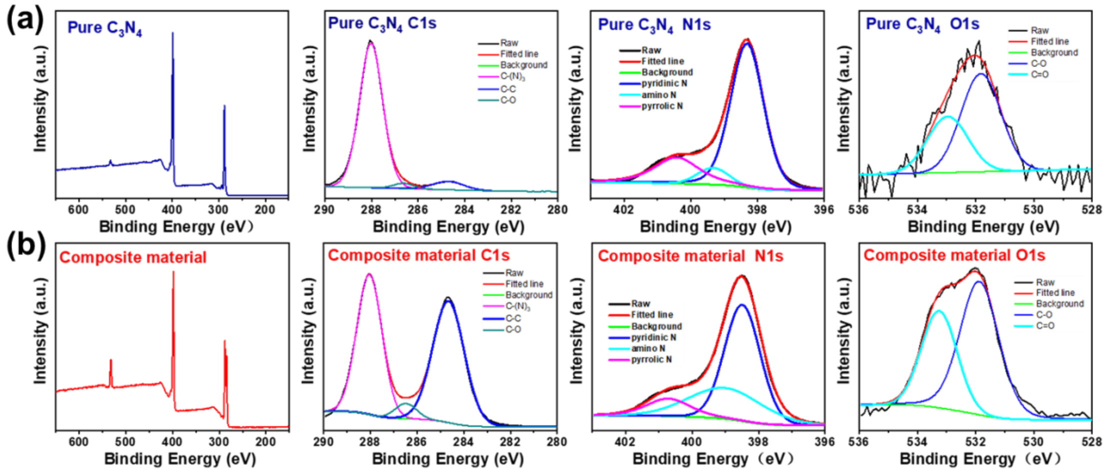

Figure S3. XPS survey spectrum of the **(a)** pure C_3N_4, and **(b)** composite material

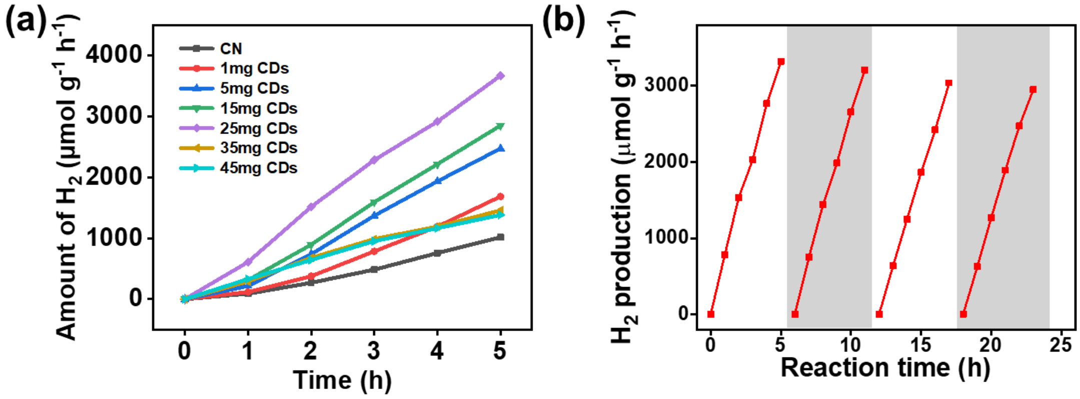

Figure S4. (a) Photocatalytic H_2 production of 2,4-CN with different content CDs, loading 1.0 wt% Pt in the 10 vol% triethanolamine (TEOA) under light irradiation ($\lambda > 420$ nm). **(b)** the stability experiment of the best composite material

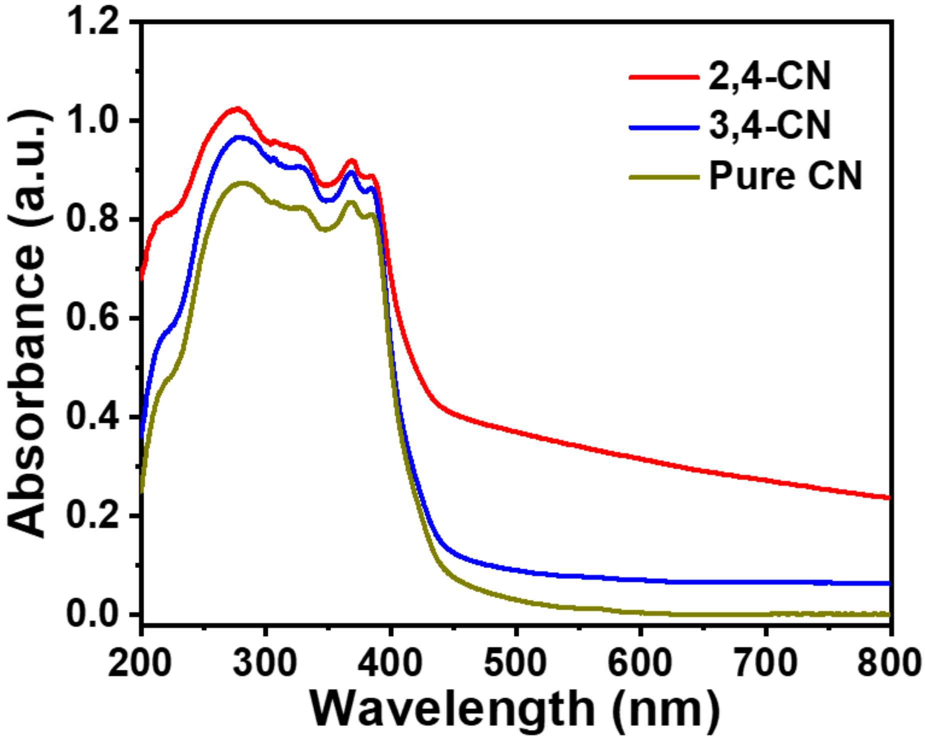

Figure S5. DRS UV-vis spectra of pure CN, 2,4- and 3,4-CN.

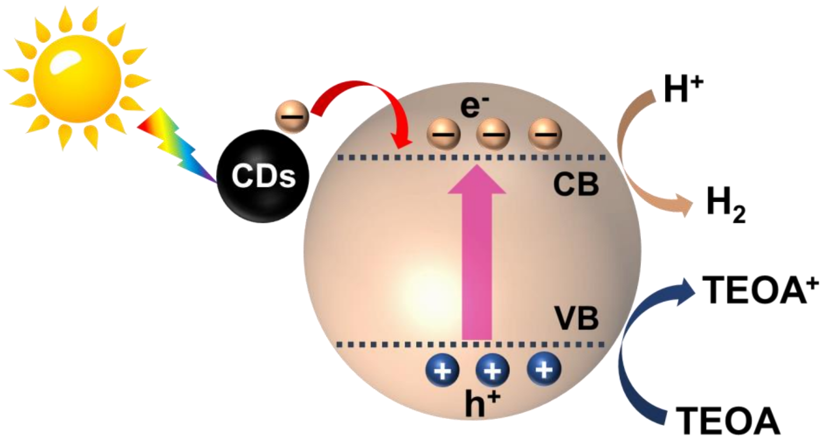

Figure S6. The schematic diagram of the composite photocatalytic hydrogen generation mechanism

Table S1. The photocatalysis hydrogen production efficiency comparative table of CDs composition

Composite /heterojunction	Method of synthesis	Hydrogen production efficiency	Light source	References
CNNS/CQDs	Solvothermal method	116.1 μmol h^{-1}, 3 times higher than pure CNN	300 W Xe arc lamp	[1]
g-C_3N_4/TiO_2/ CQDs/Au	Solvothermal method	5.69 mmol g^{-1} h^{-1}, 6.2 times higher than pure g-C_3N_4	500 W Xe lamp	[2]
ZnO/C_3N_4 QDs	Dip-coating method	2.29 times higher than pure ZnO		[3]
OH–CQDs/CNF-X	Solvothermal approach	357.98 μmol g^{-1}	300 W Xe lamp	[4]
CQD/CdS	hydrothermal and stirring	~309 mmol g^{-1} h^{-1}	visible light	[5]
CD-C_3N_4 composite	electrochemical hydrothermal	~8.4 mmol h^{-1}	300 W Xe lamp	[6]
CQD/HCN Nanocomposites	furnace (500 °C)	382 μmol g^{-1} h^{-1}	500 W xenon arc lamp	[7]
S, N-CD/g-C_3N_4 composite	Hydrothermal Solvothermal	832 μmol g^{-1} h^{-1}	300 W Xe lamp	[8]
CQDs/2D g-C_3N_4	Hydrothermal	183 μmol h^{-1}; 48.1 μmol h−1	UV: 365 nm; Vis: 420 nm 3 W	[9]
CQDs/2D TiO_2	Hydrothermal	79.3 μmol g^{-1} h^{-1}	500 W Hg lamp, λ not specified	[10]
CQDs/2D g-C_3N_4/2D TiO_2	Oven drying	6.497 μmol g^{-1} h^{-1}	Vis: >400, 300 W Xe lamp	[11]
2,4-CDs@C_3N_4	homogeneous thermal pyrolysis	733.8μmol g^{-1} h^{-1}, 3.6 times higher than pure CN	300 W Xe lamp	Our work

References

1. Sun, C.; Xu, Q.; Xie, Y.; Ling, Y.; Jiao, J.; Zhu, H.; Zhao, J.; Liu, X.; Hu, B. Zhou, D. High-efficient One-pot Synthesis of Carbon Quantum Dots Decorating Bi_2MoO_6 Nanosheets Heterostructure with Enhanced Visible-light Photocatalytic Properties. J. Alloy. Compd. 2017, 723, 333-344.
2. Tian, J.; Leng, Y.; Zhao, Z.; Xia, Y.; Sang, Y.; Hao, P.; Zhan, J.; Li, M. Liu, H. Carbon Quantum dots/hydrogenated TiO_2 Nanobelt Heterostructures and Their Broad Spectrum Photocatalytic Properties under UV, Visible, and Near-infrared Irradiation. Nano Energy 2015, 11, 419-427.
3. Zhang, Z.; Zheng, T.; Xu, J.; Zeng, H. Zhang, N. Carbon Quantum Dots/Bi_2WO_6 Composites for Efficient Photocatalytic Pollutant Degradation and Hydrogen Evolution. Nano 2017, 12, 1750082.
4. Li, S.; Hu, S.; Jiang, W.; Liu, Y.; Zhou, Y.; Liu, J. Wang, Z. Facile Synthesis of Cerium Oxide Nanoparticles Decorated Flower-like Bismuth Molybdate for Enhanced Photocatalytic Activity toward Organic Pollutant

Degradation. J. Colloid. Interf. Sci. 2018, 530, 171-178.

5. Gogoi, D.; Koyani, R.; Golder, A.K. Peela, N.R. Enhanced Photocatalytic Hydrogen Evolution using Green Carbon Quantum Dots Modified 1-D CdS Nanowires under Visible Light Irradiation. Sol. Energy 2020, 208, 966-977.
6. Liu, J.; Liu, Y.; Liu, N.; Han, Y.; Zhang, X.; Huang, H.; Lifshitz, Y.; Lee, S.-T.; Zhong, J. Kang, Z. Metal-free Efficient Photocatalyst for Stable Visible Water Splitting via a Two-electron Pathway. Science 2015, 347, 970-974.
7. Li, L.; Zhu, X. Enhanced Photocatalytic Hydrogen Evolution of Carbon Quantum Dot Modified 1D Protonated Nanorods of Graphitic Carbon Nitride. ACS Appl. Nano Mater. 2018, 1, 5337-5344.
8. Xie, Z.; Yu, S.; Fan, X.-B.; Wei, S.; Yu, L.; Zhong, Y.; Gao, X.-W.; Wu, F. Zhou, Y. Wavelength-sensitive Photocatalytic H_2 Evolution from H_2S Splitting over g-C_3N_4 with S,N-codoped Carbon Dots as The Photosensitizer. J. Energy Chem. 2021, 52, 234-242.
9. Naseri, A.; Samadi, M.; Pourjavadi, A.; Moshfegh, A.Z. Ramakrishna, S. Graphitic Carbon Nitride (g-C_3N_4)-based Photocatalysts for Solar Hydrogen Generation: Recent Advances and Future Development Directions. J. Mater. Chem. A 2017, 5, 23406-23433.
10. Wang, Q.; Yang, Z. Industrial Water Pollution, Water Environment Treatment, and Health Risks in China. Environ. Pollut. 2016, 218, 358-365.
11. Ajmal, A.; Majeed, I.; Malik, R.N.; Idriss, H. Nadeem, M.A. Principles and ,Mechanisms of Photocatalytic Dye Degradation on TiO_2 Based Photocatalysts: a Comparative Overview. RSC Adv. 2014, 4, 37003-37026.
